# Genome-wide analysis of DNA replication timing in single cells: Yes! We’re all individuals

**DOI:** 10.1186/s13059-019-1719-y

**Published:** 2019-05-30

**Authors:** Anne D. Donaldson, Conrad A. Nieduszynski

**Affiliations:** 1 Institute of Medical Sciences, University of Aberdeen, Foresterhill, Aberdeen, AB25 2ZD UK; 2 Sir William Dunn School of Pathology, University of Oxford, South Parks Road, Oxford, OX1 3RE UK

## Abstract

Recent studies have accomplished the extraordinary feat of measuring the exact status of DNA replication in individual cells. We outline how these studies have revealed surprising uniformity in how cells replicate their DNA, and consider the implications of this remarkable technological advance.

## Introduction

The DNA of eukaryotic genomes is replicated in a characteristic temporal order. In mammalian cells, clusters of replication origins initiate synchronously, leading to regions of chromosomal DNA that replicate at a particular time during S-phase; these are referred to as replication-timing domains. The pattern of replication-timing domains leads to a genome-wide replication timing profile that, although generally fairly stable for the genome of a particular organism, does show some differences that are dependent upon cell type and developmental status. In particular, genomic loci have been identified that undergo clear transitions in their replication timing during differentiation, sometimes correlating with the expression status of the genes they contain. Early-replicating domains tend to be euchromatic and enriched for marks of open and active chromatin, whereas late-replicating domains are enriched for closed, inactive heterochromatic marks. One special case is the X chromosome, where the inactive X of mammalian females becomes almost entirely late-replicating as its transcription is shut down. There are multiple links between replication timing and genome stability: replication time correlates with mutation rate and timing profiles are often disrupted in cancer cells [1], potentially contributing to chromosomal breakage, translocations and aneuploidy.

In the past two decades, many studies have analyzed replication timing genome-wide [2, 3], most recently using high-throughput sequencing to detect either newly replicated DNA (often after BrdU-labelling then immunoprecipitation) or the doubling in copy number that occurs as the DNA is replicated. Such methods have mostly been used to analyze cell populations, with the resulting data representing the average replication time for each genomic sequence across all cells. As a result, it has been difficult to estimate the heterogeneity in replication time—either variation at a specific locus between different cells of a population or variation between different loci in a single cell that share the same average replication time. However, single-cell DNA sequencing techniques have now made possible the remarkable feat of analyzing the replication status of an individual cell [4–6]. The latest of these investigations, from the Hiratani lab [7], presents a particularly interesting and thorough analysis of replication dynamics that is based on the analysis of individual cells, providing the most detailed description yet of the extent of ‘between-cell‘ and ‘within-cell’ variability in the replication-timing program.

## Single-cell analysis of replication timing confirmed the stability of the replication program

**Fig. 1:**
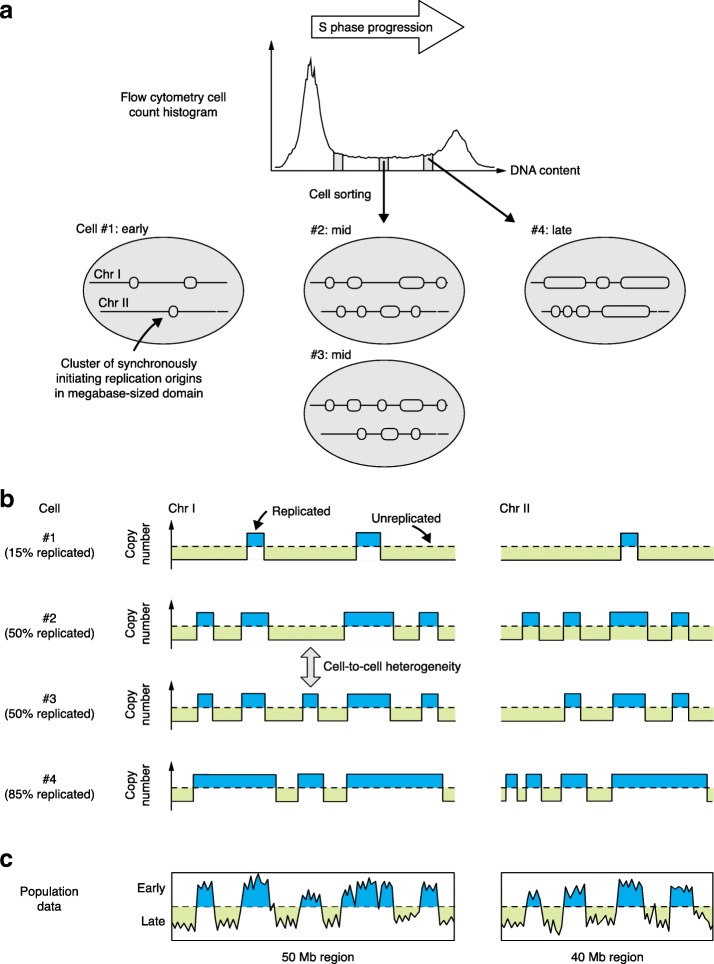
Overview of the procedure used by Takahashi et al. [7] to analyze the replication status of single cells from mouse-derived cell lines. **a** Individual cells in early, mid or late S-phase were sorted on the basis of their DNA content. The cartoons of cells below illustrate the approximate extent of replication for two chromosome segments in four different cells, cell #1 (early S-phase), cells #2 and #3 (mid S-phase), and cell #4 (late S-phase). Note that these chromosome cartoons illustrate segments of about 50 Mb, with replicated regions of around 1–10 Mb in length that correspond to clusters of activated origins rather than to individual origin sites. **b** Principle of replication status analysis in single cells. Sequences are assigned as replicated (Copy number 2; *blue fill*) or not (Copy number 1; *green fill*) on the basis of their representation in the high-throughput sequencing analysis of each single cell, as illustrated in the stylized plots shown for each cell. The sequence data also allow assignment of the percentage of the genome that is replicated in each cell (shown on the left). **c** Plots illustrate the replication timing curves that would be obtained for the same chromosome segments using traditional genome-wide replication timing analysis in a large cell population (such as population Repli-seq as described in [7]); the results are expected to resemble mid-S-phase copy number plots (i.e. cells #2 and #3) most closely

The approach taken by Takahashi et al. [7] was to isolate single mid-S-phase cells by flow cytometry, then to extract and amplify the DNA from these individual cells for next-generation sequencing (Fig. 1a). Analysis of sequence ‘copy number’ in the results—that is, comparison of the relative representation of all sequences with their representation in G_1_-phase cells—then revealed which sequences had been replicated in the particular cell being assessed (Fig. 1b, regions filled blue). Validating the approach, the plots obtained in this way show replicated DNA patterns that closely resemble those from more traditional analyses of replication timing (Fig. 1c). Comparison of the single-cell data with those obtained from a large S-phase cell population (whose DNA had not been amplified) provided reassurance that the PCR amplification necessary in the single-cell procedure did not bias the results.

Plotting results from multiple single cells produced a pleasingly graphic comparison of the replication profiles, highlighting similarities and any differences between cells. Indeed, cells of the same type that were isolated at the same S-phase stage had generally replicated largely the same set of sequences (Fig. 1b, cells #2 and #3). One limitation of the approach is that each individual dataset provides a static snapshot of the situation in a particular cell at the point at which it was isolated. The authors were, however, able to monitor the replication of each particular locus throughout S-phase by carrying out the same analysis for cells isolated at different stages of S-phase, then vertically stacking the results from individual cells at successively more advanced stages of genomic replication (as illustrated in Fig. 1b). Moreover, the authors were able to measure the interval between the earliest and latest replication of any particular locus. In agreement with an earlier, similar study [6], these ‘earliest to latest replication time’ intervals were generally fairly narrow, meaning that loci replicate at a fairly consistent time in different cells. For most sequences, replication consistently occurs within about 1 h on either side of their average replication time, within an S-phase that lasts about 10 h overall. Nonetheless, some specific sequences did show greater heterogeneity in replication time.

## What does the analysis reveal?

Takahashi et al. [7] used their procedure to carry out a set of long-imagined experiments. As the replication program is known to change during cellular differentiation, their first experiment was to compare the replication profiles of single cells isolated from naïve and differentiated mouse embryonic stem cell (mESC) lines. Satisfyingly, the single-cell replication profiles were similar throughout most of the genome, but differences were evident at regions where the replication program was already known to be affected by differentiation, including regions undergoing both early to late and late to early developmental transitions.

One question concerning the ‘within-cell’ variability of the replication-timing program was the extent to which the loci of homologous chromosomes differ in replication timing. To address this issue, Takahashi et al. [7] used mESCs from a cross of distantly related mouse parents, in which frequent sequence differences permit the assignment of sequence reads to one or other chromosome, allowing the generation of ‘haplotype-resolved’ data that report separately on the replication status of each chromosome in a homologous pair. Mostly, homologous autosomal chromosomes showed similar replication timing. In those regions where differences were seen in the replication timing of different haplotypes in naïve mESCs, these differences tended to be fairly small and to become lost upon differentiation. At sites where there were haplotype differences in replication timing (i.e., asynchronously replicating loci) and also allelic differences in transcriptional expression, there was a strong tendency for the changes to be coordinated: the earlier-replicating allele was usually the more strongly expressed.

The naïve-to-differentiated mESC cell transition analyzed by Takahashi et al. [7] covered the step of X inactivation, and the haplotype-resolved replication timing data also elegantly allowed clear visualization of the transition to late replication of the inactive X chromosome during differentiation. One limitation of the methodology of this particular experiment was that only mid-S-phase cells were analyzed, so the results only showed that the inactive X chromosome was not yet replicated at mid-S-phase and provided no information on exactly how late it would replicate or how synchronously. A more detailed analysis of cells from a later stage of replication would shed useful light on this issue, which highlights the importance of sampling the S-phase period encompassing the replication events that are of particular interest in order to obtain the best information in this type of analysis.

The main message from the single-cell analyses is the stability of the replication program, although some interesting differences did emerge from the analyses carried out by Takahashi et al. [7]. In particular, although they found fairly limited variation in the replication times of most loci (typically about an hour) through the main part of S-phase, they observed even less variability in replication time when they examined sequences that were duplicated at the beginning or end of S-phase. This effect was not observed in the analysis by Dileep and Gilbert [6], and whether the different findings reflect differences in the sampling or data-analysis procedures remains to be seen. Takahashi et al. [7] further found that prior to differentiation, developmentally regulated genes appear to show greater heterogeneity in their replication timing than constitutively early-replicating genes. The authors point out that developmentally regulated genes also show less strict subnuclear compartmentation, suggesting the interesting possibility that such genes occupy a particularly malleable chromatin environment. Related to this point, both the Takahashi et al. [7] study and the Dileep and Gilbert [6] study found a close correlation between replication timing and the compartmental organization identified by Hi-C investigations, a relationship that was not unexpected given the links between timing and chromatin status.

## Potential of single-cell replication mapping

The most impressive aspect of these single-cell replication-timing studies stems from the remarkable depth, accuracy and richness of the information that they provide. In many respects, the findings to date confirm expectations from population studies of the replication program: (i) heterogeneity between cells and between homologous chromosomes does exist but is limited so that sequences almost always replicate close to their scheduled time; (ii) there are developmental differences in replication timing; and (iii) replication timing correlates with euchromatic or heterochromatin status and with subnuclear chromosome organization. The main excitement lies in what this technology will permit. Several factors are known or suspected to control the replication-timing program, but their precise impact on specific sites and replication-origin types remains unclear, partly because population methods have not permitted exact effects to be resolved [8]. When a derailed replication has been observed, it has been difficult to distinguish between the general randomization of the replication program and effects on specific types of chromosome domain. Now, single-cell analysis of replication should allow such issues to be resolved clearly, and so can be expected to provide a dramatic advancement of our knowledge of how replication is controlled. Moreover, the technology could allow a clear understanding of infrequent events. For example, it appears that DNA replication sometimes fails to complete during interphase, resulting in occasional use of a more error-prone mitotic DNA repair synthesis (MiDAS) pathway [9]. Given the unpredictability of their locations, sites of incomplete DNA replication could potentially be analyzed by sequencing single post-S-phase cells. Similarly, we can expect single-cell approaches to deliver a much more detailed understanding of the events that occur when replication is interrupted, such as how replication-inhibiting drugs impact on S-phase progress during and after treatment, and to elucidate other clinically relevant effects, such as where and how uniformly replication profiles are changed in cancer cells.

What this analysis does not yet provide is detailed initiation site information: because origins fire in clusters, new bubble structures rapidly merge, preventing the actual initiation sites from being detected by a snapshot approach. In addition, pinpointing initiation events that could be ‘caught in the act’ would require higher resolution than that presented to date. However, another remarkable new technology, ultra-long nanopore sequencing identification of nascent DNA, is set to advance our understanding of replication initiation site specification greatly in the near future [10]. Single molecule analysis of nascent DNA will finally identify the exact sites at which replication initiates in mammalian cells, and will shed light on how origin clusters are coordinately regulated. Combining a newly accurate view of replication initiation with single-cell analysis of replication timing can be expected to provide a greatly improved understanding of replication dynamics and of the control of replication in mammalian cells.

## Conclusion

Overall, recent studies have provided an ultra high-resolution view of how cells progress through the replication program. By parsing within-cell variability from population effects, the combination of single-cell and single-molecule approaches to analyze replication holds enormous potential. The resolution and accuracy now offered by such methods will open a new and exciting era in understanding how cells replicate their genomes.

## Abbreviation

mESC: Mouse embryonic stem cell
